# Population Size Estimations Among Hidden Populations Using Respondent-Driven Sampling Surveys: Case Studies From Armenia

**DOI:** 10.2196/12034

**Published:** 2019-03-14

**Authors:** Katherine R McLaughlin, Lisa G Johnston, Laura J Gamble, Trdat Grigoryan, Arshak Papoyan, Samvel Grigoryan

**Affiliations:** 1 Department of Statistics Oregon State University Corvallis, OR United States; 2 5% Initiative Expertise France Paris France; 3 National Center for AIDS Prevention Yerevan Armenia

**Keywords:** population size estimation, respondent-driven sampling, men who have sex with men, female sex workers, people who inject drugs, Armenia

## Abstract

**Background:**

Estimates of the sizes of hidden populations, including female sex workers (FSW), men who have sex with men (MSM), and people who inject drugs (PWID), are essential for understanding the magnitude of vulnerabilities, health care needs, risk behaviors, and HIV and other infections.

**Objective:**

This article advances the successive sampling-population size estimation (SS-PSE) method by examining the performance of a modification allowing visibility to be jointly modeled with population size in the context of 15 datasets. Datasets are from respondent-driven sampling (RDS) surveys of FSW, MSM, and PWID from three cities in Armenia. We compare and evaluate the accuracy of our imputed visibility population size estimates to those found for the same populations through other unpublished methods. We then suggest questions that are useful for eliciting information needed to compute SS-PSE and provide guidelines and caveats to improve the implementation of SS-PSE for real data.

**Methods:**

SS-PSE approximates the RDS sampling mechanism via the successive sampling model and uses the order of selection of the sample to provide information on the distribution of network sizes over the population members. We incorporate visibility imputation, a measure of a person’s propensity to participate in the study, given that inclusion probabilities for RDS are unknown and social network sizes, often used as a proxy for inclusion probability, are subject to measurement errors from self-reported study data.

**Results:**

FSW in Yerevan (2012, 2016) and Vanadzor (2016) as well as PWID in Yerevan (2014), Gyumri (2016), and Vanadzor (2016) had great fits with prior estimations. The MSM populations in all three cities had inconsistencies with expert prior values. The maximum low prior value was larger than the minimum high prior value, making a great fit impossible. One possible explanation is the inclusion of transgender individuals in the MSM populations during these studies. There could be differences between what experts perceive as the size of the population, based on who is an eligible member of that population, and what members of the population perceive. There could also be inconsistencies among different study participants, as some may include transgender individuals in their accounting of personal network size, while others may not. Because of these difficulties, the transgender population was split apart from the MSM population for the 2018 study.

**Conclusions:**

Prior estimations from expert opinions may not always be accurate. RDS surveys should be assessed to ensure that they have met all of the assumptions, that variables have reached convergence, and that the network structure of the population does not have bottlenecks. We recommend that SS-PSE be used in conjunction with other population size estimations commonly used in RDS, as well as results of other years of SS-PSE, to ensure generation of the most accurate size estimation.

## Introduction

Having accurate estimates of the sizes of hidden populations, including female sex workers (FSW), men who have sex with men (MSM), and people who inject drugs (PWID), are essential for understanding the magnitude of vulnerabilities, health care needs, risk behaviors, and HIV and other infections. In addition, population size estimations (PSEs) are used to inform resource allocation to develop programs to support sexual health and well-being, counseling and treatment for drug use, to advance social and economic justice, and to respond to and monitor critical health needs and epidemics. However, measuring a hidden population is extremely challenging and current methods contain numerous biases [1-5]. Given the importance of measuring the sizes of hidden and vulnerable populations, the advancement and continued critical review of current methods are needed [2,6].

Currently, many PSEs of FSW, MSM, and PWID are conducted in conjunction with HIV biobehavioral surveys (BBS) using respondent-driven sampling (RDS) [1-5,7-9]. These surveys are routinely implemented to measure the prevalence of sexual risks, drug use, HIV testing and knowledge, stigma and discrimination, and HIV and other infections among key populations at higher risk of HIV exposure.

RDS is a probability-based sampling method which, when implemented and analyzed correctly, can yield findings representing the network of the population sampled [10-13]. Sampling begins with a convenience sample of well-networked population members, referred to as *seeds*. Seeds enroll, complete the survey and biological specimen collection, and are provided a fixed number of coupons to recruit members from their social network. All participants provide a measurement of their social network size, or degree, which is the number of people they know, who know them, that are in their social network. For BBS, social networks are described as groups of people who know each other and engage in common behaviors, such as injecting drugs, having anal sex, or exchanging sex for money or goods during a specified time period (eg, 6 months). Coupons, which contain a unique number to manage peer-to-peer recruitment, allow participants to remain anonymous, making it especially acceptable to populations that are stigmatized or practice illegal behaviors. Sampling should result in long recruitment chains, whereby the final sample is not biased by the initial convenience sample of seeds. Data collected using RDS methods are adjusted based on each participant’s social network size and other covariates when making inferences about the population to account for the complex sampling process. The intuitive reasoning is that individuals with larger social network sizes are more likely to be sampled, and be sampled earlier in the RDS process, so their responses need to be weighted accordingly.

One of the PSE methods being commonly used in conjunction with RDS surveys is successive sampling-population size estimation (SS-PSE) [4,5,7,9,14,15], which relies on the successive sampling model for the RDS sampling process [16]. Unlike other PSE methods that use two or more sources of data, such as object and service multipliers and capture-recapture, SS-PSE can be used with data from just one RDS study. In SS-PSE, the order of enrollment and network size of each participant are used to estimate the distribution of population network sizes and the depletion of network size over the study period is used to model the overall population size. More details will be provided in the Methods section.

**Figure 1:**
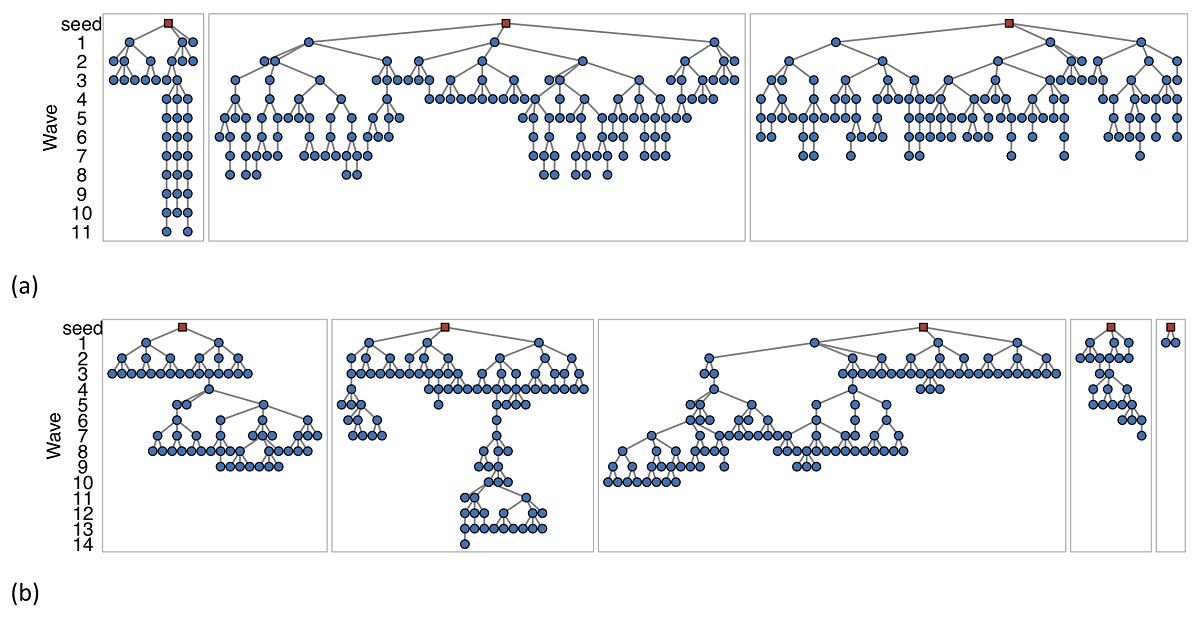
Respondent-driven sampling (RDS) recruitment chains for selected populations: (a) female sex workers (FSW), Yerevan 2016 and (b) men who have sex with men (MSM), Yerevan 2014. Seeds are indicated by red squares and the waves of recruitment are shown vertically.

This article describes the use of SS-PSE in three rounds of BBS, conducted in 2012, 2014, and 2016, among FSW, MSM, and PWID in three cities in Armenia: Yerevan, the capital city (2016 population: 467,087 females and 373,903 males, aged 18 years or older); Gyumri, the second-largest city, located in the northwest of Armenia (2016 population: 49,482 females and 41,535 males, aged 18 years or older); and Vanadzor, the third-largest city, located in the north of Armenia (2016 population: 26,052 females and 28,962 males, aged 18 years or older) [17]. Roughly 43% of the country’s population live in these areas. RDS recruitment chains for selected populations are shown in Figure 1 and the complete set are provided in Multimedia Appendix 1. We advance the SS-PSE methodology by examining the performance of a modification allowing visibility, a measure of a person’s propensity to participate in the study, to be jointly modeled with population size in the context of 15 datasets of FSW, MSM, and PWID populations from Armenia. Visibility [18] is modeled because inclusion probabilities for RDS are unknown and social network sizes, often used as a proxy for inclusion probability, are subject to biases and measurement errors from self-reported study data. For example, self-reported social network sizes may be an inaccurate measure of inclusion probability due to heaping or rounding [16]; intentional misreporting, perhaps to minimize one’s connection to a stigmatized population [19-21]; and unintentional misreporting, perhaps due to memory recall bias [22-24]. We compare and evaluate the quality of our imputed visibility PSEs to those found for the same populations through other unpublished methods. In addition, we provide guidelines and caveats to improve the implementation of SS-PSE for real data.

## Methods

### Survey

Standard RDS methods were used to recruit FSW, MSM, and PWID in 2012, 2014, and 2016 in Yerevan as well as in 2016 in Gyumri and Vanadzor [10]. Network size questions were structured based on the eligibility criteria of each sampled population: FSW were women who received money in exchange for sexual intercourse in the previous three months; MSM were men, including transgender women in 2012, 2014, and 2016, who had anal sex with another man in the previous 12 months; and PWID were people who injected drugs for nonmedical purposes in the previous three months. All participants were 18 years of age or older and residents of the survey location. Seeds or persons with a valid coupon who presented to a survey location were screened for eligibility and underwent informed consent. No one refused to enroll, despite having to consent to both the biological and behavioral parts of the survey. Enrollees were then interviewed by a trained interviewer, provided HIV pretest counseling, and underwent a venous blood draw for laboratory testing for HIV and other infections. Following the blood draw, each respondent received a set number of coupons—no more than three—along with recruitment instructions on how to recruit eligible peers. The different number of coupons to distribute and different target sample sizes—100 in Gyumri and Vanadzor and 300 in Yerevan—reflect differences in population size and connectedness, as well as anticipated speed of recruitment, identified during formative research. To maintain respondents’ confidentiality, unique identification codes were used to link behavioral and biological data and to track who recruited whom. Respondents received primary compensation of AMD 4000 (Armenian Dram) in 2012 and 2014 and AMD 3500 in 2016, or slightly over US $7 using 2016 conversion rates, for enrollment and completion of the survey. Respondents received an additional secondary compensation—AMD 2000 in 2012 and 2014 and AMD 1600 in 2016—for each peer they recruited who enrolled and completed the survey.

The network size question is crucial to RDS studies as a proxy for a person’s propensity to be included in the sample. Participants were asked how many individuals they know who meet the study eligibility requirements and then, as a follow-up, how many of them they have seen in the previous month. An individual’s network size is considered to be the second, more restrictive, of these numbers. For example, the precise question for FSW in Vanadzor was “How many women do you know, whom also know you, are 18 years of age and older, are living in Yerevan, and have exchanged vaginal or anal sex for money or other reward? How many of them have you seen in the past month?”

### Successive Sampling-Population Size Estimation and Visibility Imputation

Population size estimations were conducted using SS-PSE [25,26]. The approach approximates the RDS sampling mechanism via the successive sampling model of Gile [27] and uses the order of selection of the sample to provide information on the distribution of network sizes over the population members. SS-PSE uses a Bayesian framework, treating the population size N as unknown, but with a specified prior distribution. The SS-PSE framework allows for the incorporation of prior belief about the population size, which is often available via expert knowledge or PSEs from other sources, such as enumeration through mapping, network scale-up, multiplier, or capture-recapture methods [7]. The population unit sizes are treated as independent and identically distributed samples generated from a superpopulation model based on some unknown distribution. This setup is common in model-based sampling theory [28]; in it, the unit values of the finite population are conceived of as a random sample from an infinite population or superpopulation rather than fixed, but are unknown. We observe a subset n<N of members of the population in our sample, as well as the self-reported degree for each individual and order of participation (ie, enrollment date).

**Figure 2:**
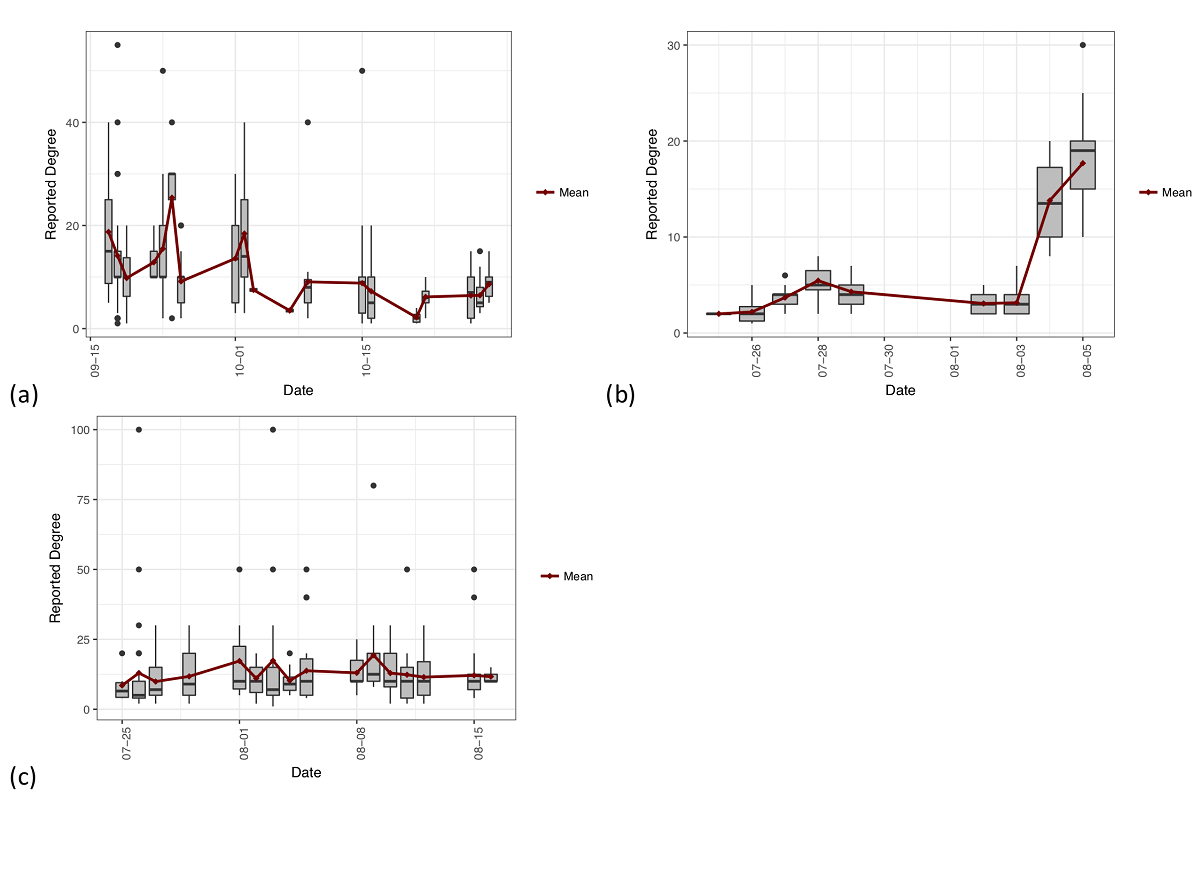
Plots of enrollment date versus self-reported network size for selected populations: (a) female sex workers (FSW), Yerevan 2014, showing a depletion in mean reported degree over the study period; (b) men who have sex with men (MSM), Vanadzor 2016, showing an increasing trend over the study period; and (c) FSW, Yerevan 2016, showing a constant trend over the study period. Note that the magnitude of trends is not comparable across plots due to different reported degree values for the different populations.

The successive sampling model assumes that individuals with a higher degree are more likely to be recruited earlier in the RDS process, since they are more connected and easily accessible in the social network. Thus, if there are fewer large-degree individuals in later waves than earlier waves, this suggests a depletion of members of the population and a large sample fraction; the population is likely not much larger than the sample. However, if the reported degrees stay roughly the same across recruitment waves, the sample size is likely a smaller portion of the population. If the reported degrees increase notably across waves, this may be an indication that the RDS recruitment process is not operating as expected and would merit caution when interpreting the results of various estimators. Figure 2 shows plots of enrollment date versus reported degree for selected populations. Panel (a) demonstrates a situation in which few large-degree individuals are observed in the later waves and the overall trend is slowly decreasing. Panel (b) shows a strongly increasing trend, which indicates that the SS-PSE method may not perform well for this population. Panel (c) shows a relatively constant degree across waves, with some large-degree individuals still present in the later waves of the sample. These types of exploratory plots aid in understanding how RDS recruitment dynamics affect SS-PSE estimates and can alert us to possible violations of sampling assumptions.

The original SS-PSE method relies on self-reported network sizes. However, these values are subject to bias due to heaping or rounding and both intentional and unintentional misreporting; additionally, they may contain missing or impossibly low or high values [11]. We therefore use a modified version of SS-PSE that jointly models the visibility of each individual using a measurement error model [18]. Visibility is viewed as an adjusted or underlying degree that attempts to account for the aforementioned issues that arise from self-reports. We use a Conway-Maxwell-Poisson measurement error model that allows for the proportional inflation of the self-reported degree relative to the visibility and for relative error of the self-reported degrees around this inflated value. Computationally, this modification adds two additional components that need to be estimated during each step of the SS-PSE Markov chain Monte Carlo algorithm, but the outputs from the method are the same.

Imputed visibility SS-PSE is a Bayesian method, where information about unknown parameters is expressed through probability distributions over their possible values. Thus, the resulting estimates take the form of a distribution called the posterior distribution. We estimate the posterior distribution for the population size N, given our prior belief about the population size and observed data. The prior information used for each of the imputed visibility SS-PSE models of the 15 Armenian datasets was a median, obtained as the average of two medians for that population and city provided by local experts in 2016 through a consensus and extrapolation led by the second author (LGJ) [29]. Local experts included representatives from the National Center for AIDS Prevention, the Joint United Nations Programme on HIV/AIDS (UNAIDS), the Global Fund, and several governmental and nongovernmental organizations working directly with the populations. Local experts believe the population sizes of FSW, MSM, and PWID remained relatively constant over the period from 2012 to 2016, as reported by the Statistical Committee of the Republic of Armenia [17]; thus, we used the same prior medians for each of the three years for each population in Yerevan. To examine sensitivity to this choice of prior medians, we also fit each imputed visibility SS-PSE model using each of the two expert prior medians separately. Because population size distributions are skewed, we used the median of the posterior distribution as a point estimate and 90% credible intervals to express uncertainty about the estimate. In addition to individual PSEs for each dataset, we also compared the estimates for the Yerevan populations across the 2012, 2014, and 2016 surveys. We assessed the trend of the estimates, considering their overall variability, using mirrored plots of the posterior distributions. Imputed visibility SS-PSE estimates were performed using the *posteriorsize* function in the *sspse* package, version 0.8, for the R programming language (The R Foundation) [18].

## Results

### Population Size Estimates

We applied the imputed visibility SS-PSE method to 15 datasets of FSW, MSM, and PWID populations from Armenia. Table 1 reports the prior values and quantiles of the posterior distribution for population size from each of the populations. Reference values provided by local experts are shown as well, where the expert median is the value used as the prior median in the imputed visibility SS-PSE model. The expert low and high values are, respectively, the minimum of two expert values provided for the smallest that the population size could be and the maximum of two expert values provided for the largest that the population size could be. These numbers were not used in the estimation procedure, but are used to assess the model’s goodness of fit and plausibility of the PSE.

**Table 1:** Prior values and quantiles of the posterior distribution for population size obtained from imputed visibility SS-PSE (successive sampling-population size estimation) estimates from 15 datasets of female sex workers (FSW), men who have sex with men (MSM), and people who inject drugs (PWID) populations in Armenia.

Population	Expert values, n			Posterior, n					
	Low^a^	Median (prior)^b^	High^a^	5%	25%	Median	75%	95%	Assessment^c^
FSW, Yerevan 2012	1500	3143	9900	1243	2235	3734	6975	16,599	Great
FSW, Yerevan 2014	1500	3143	9900	397	421	445	469	542	Bad
FSW, Yerevan 2016	1500	3143	9900	784	1340	2090	4169	12,924	Okay
FSW, Gyumri 2016	165	351	1089	126	152	171	217	340	Okay
FSW, Vanadzor 2016	115	239	759	133	164	205	280	551	Great
MSM, Yerevan 2012	2420	4202	6667	943	1550	2407	4335	14,296	Bad
MSM, Yerevan 2014	2420	4202	6667	836	1407	2299	4477	17,973	Bad
MSM, Yerevan 2016	2420	4202	6667	871	1121	1550	2264	4870	Bad
MSM, Gyumri 2016	176	306	485	127	176	249	409	974	Okay
MSM, Vanadzor 2016	123	214	339	323	506	666	836	993	Bad
PWID, Yerevan 2012	1667	5842	14,473	1196	2041	3236	5823	17,117	Good
PWID, Yerevan 2014	1667	5842	14,473	1196	2091	3435	3569	19,008	Good
PWID, Yerevan 2016	1667	5842	14,473	698	947	1245	2091	6072	Bad
PWID, Gyumri 2016	167	584	1446	197	354	596	1141	2960	Great
PWID, Vanadzor 2016	117	409	1013	201	318	477	812	1775	Great

^a^The prior low and high values are, respectively, the minimum of the expert prior lows and the maximum of the expert prior highs.

^b^The prior median is the average of the expert prior medians.

^c^The *Assessment* column describes how well the estimate aligned with expert knowledge, based on low and high values provided by experts that were not used in the size estimation model.

**Figure 3:**
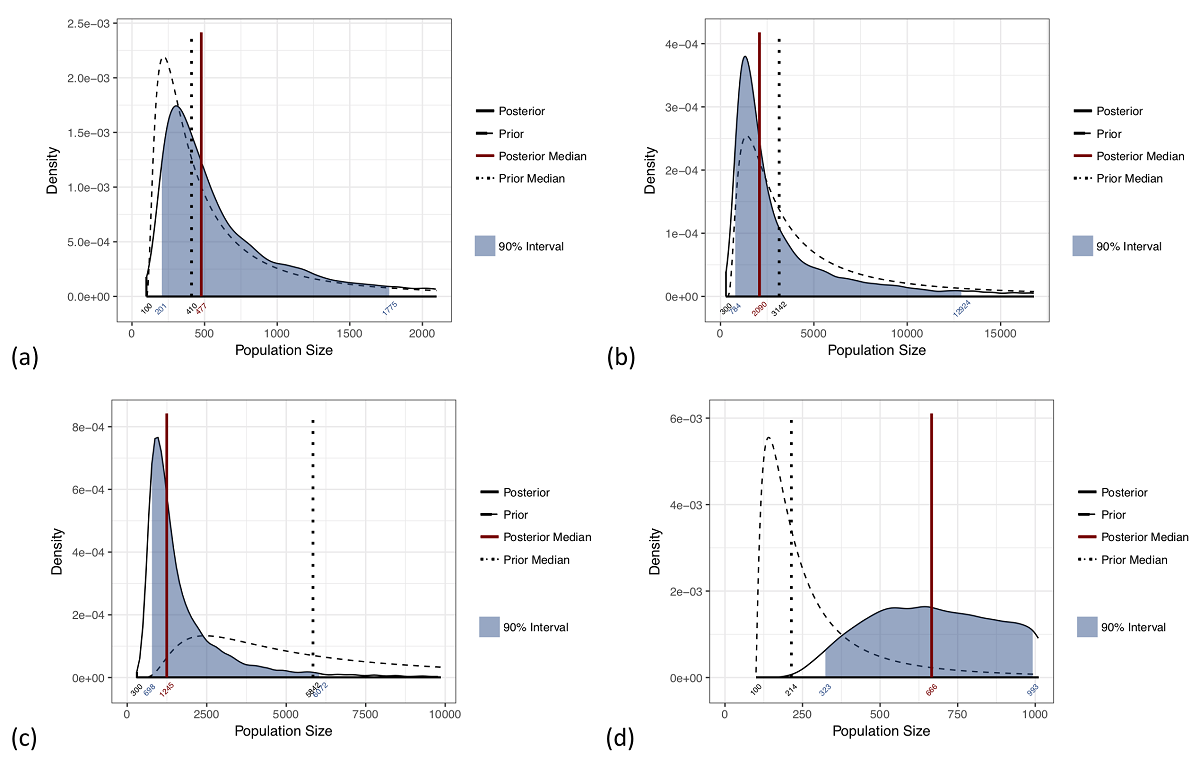
Posterior distributions for the size of selected populations: (a) people who inject drugs (PWID), Vanadzor 2016; (b) female sex workers (FSW), Yerevan 2016; (c) PWID, Yerevan 2016; and (d) men who have sex with men (MSM), Vanadzor 2016. The 90% credible interval is indicated by the shaded blue region and the posterior median by a vertical red line.

Several example posterior distributions are provided in Figure 3 and the complete set for all 15 datasets is available in Multimedia Appendix 1. The median of the posterior distribution provides a point estimate of the population size; the 90% credible interval demonstrates the uncertainty of the estimate and provides a range of likely values. The 90% credible interval is indicated on the posterior distribution plots as the shaded blue region and in Table 1 as the 5% and 95% values.

Each PSE was assessed by comparing the posterior median with the low and high values provided by experts. A *Great* fit is one where the posterior median is between the maximum expert low and the minimum expert high values; a *Good* fit is one where the posterior median is between the average expert low and the average expert high values; an *Okay* fit is one where the posterior median is between the minimum expert low and the maximum expert high values; and a *Bad* fit is one where the posterior median is either smaller than the minimum expert low or larger than the maximum expert high value. The low and high prior values shown in Table 1 represent the range used for an *Okay* fit, so if the posterior median falls between these two values, the fit will be *Okay* or better. Of the 15 populations considered, 4 (27%) size estimates were *Great*, 2 (13%) were *Good*, 3 (20%) were *Okay*, and 6 (40%) were *Bad*. Note that due to inconsistency of the expert prior medians provided for all MSM populations, *Great* fits were not possible for 5 out of the 15 (33%) datasets. A possible explanation for this is provided in the Discussion.

The posterior distributions for population size, like those shown in Figure 3, can also be used to assess SS-PSE model fit. To aid interpretation of the SS-PSE methodology, we show examples of several different-quality fits. Panel (a) PWID, Vanadzor 2016, and panel (b) FSW, Yerevan 2016, of Figure 3 both demonstrate fits that look to be of good quality because the shape of the posterior distribution is similar to the prior distribution, with a long right tail and most of the mass of the distribution near smaller values. In panel (a), the posterior median is a little larger than the prior median, evidenced by the slight right shift of the posterior distribution relative to the prior distribution. This indicates that the RDS data provided evidence that the true population size was slightly larger than the prior belief specified. Conversely, in panel (b), the posterior median is a little smaller than the prior median and the posterior distribution is shifted slightly to the left relative to the prior distribution. This indicates that the RDS data provided evidence that the true population size was slightly smaller than the prior belief specified.

Panel (c) PWID, Yerevan 2016, provides an example of a case that is more difficult to interpret. Although the shape of the posterior distribution is acceptable and does not indicate problems with convergence of the SS-PSE method, it is clear that much of the mass from the posterior distribution falls below the prior distribution. This indicates that the PSE is much smaller than the prior median specified. Upon examining these data, we did not observe RDS assumption violations. A possible explanation is that a bottleneck in the underlying social network affected recruitment, making it difficult or impossible to sample from a portion of the population. This means that, in practice, the PSE is only for a subgroup within the overall PWID population. When size estimates are much smaller than experts expect, this could be indicative of a disjoint network, bottleneck, or other reason why only a subset of the population was reachable in the sample. In this case, we advise study officials to return to the formative research study protocol to consider whether any of these scenarios were possible [11,30]. When the assessment of the SS-PSE model does not indicate convergence problems, but the estimates produced are very different from our prior beliefs, it is advisable to return to the study context and consider whether recruitment and study participation were impacted by any additional factors.

In contrast to panel (c), where the estimate was *Bad*, but the overall SS-PSE fit was acceptable, panel (d) MSM, Vanadzor 2016, provides an example of poor SS-PSE fit. The shape of the posterior distribution is much flatter than in the other examples, does not overlap with the prior distribution much, and has most of its mass on larger population sizes. The MSM, Vanadzor 2016, data show an increasing trend in network sizes over the time of the study period, as previously discussed in the Visibility Imputation section in the Methods and shown in Figure 2 (b). This is contrary to the typical RDS assumptions that high-degree individuals will be recruited earlier and that the depletion of high-degree individuals can be used to assess population size. Because the high-degree individuals were recruited toward the end of the study, the SS-PSE model estimates that the population size is actually quite a bit larger than the prior median provided. In cases such as this, where the distribution of network sizes throughout the recruitment chain does not meet the RDS assumptions, we recommend careful consideration of the data by experts to assess the RDS study. The SS-PSE results should only be used with extreme caution.

Overall, many of the point estimates tend to be lower than the expert prior median. This scenario may reflect the reality that RDS surveys may not be reaching the full hidden population, perhaps due to bottlenecks, clustering, or isolated individuals, resulting in a PSE only for the subpopulation that is reachable by RDS.

### Comparison With Other Population Size Estimations

To place the estimates obtained using imputed visibility SS-PSE in context, we compare the posterior medians to PSEs obtained using service and unique object multiplier methods and wisdom of the crowds for the nine datasets in 2016; we also compare the posterior medians to SS-PSE without visibility imputation for all 15 datasets. The service multiplier method requires two overlapping data sources, including a count of nonduplicated clients accessing a service and a probability-based survey. For these estimations, the service data were unique counts of key populations who received an HIV test between January 1 and June 30, 2016. The PSE is this count divided by the proportion who reported having an HIV test in the probability-based survey (ie, the RDS surveys, also used for the SS-PSE models). Similarly, the unique object multiplier estimate is the number of unique objects distributed to the key population divided by the proportion who reported receiving that object in the probability-based survey. The unique object distributed was a leather bracelet for all populations in 2016, given out one week prior to the start of the survey by outreach workers. Multiplier methods rely on several assumptions, including that no individual is counted more than once in each multiplier, that there is limited in-and-out-migration, that the two data sources are independent of each other, and that the probability-based survey is representative of the hidden population. Wisdom of the crowds assumes that, in aggregate, the responses of a sufficient number of key population members about the size of their population will provide a good estimate of the actual size of their population. Participants in the RDS survey were asked for their best guesstimate on the population size and the average was computed.

Table 2 compares the point estimates for the PSEs for the 15 datasets using object and service multipliers, wisdom of the crowds, SS-PSE without visibility imputation, and imputed visibility SS-PSE. The expert values are provided for reference as well. The SS-PSE estimates compare favorably to PSEs using object and service multiplier methods, which are commonly either much too small (eg, FSW, Yerevan 2016 and FSW, Gyumri 2016) or much too large (eg, PWID, Gyumri 2016 and PWID, Vanadzor 2016). Similarly, the wisdom of the crowds’ estimates generally seem much too small (eg, 26 for PWID, Gyumri 2016, when the RDS sample size was 100) or much too large (eg, 19,342 for PWID, Yerevan 2016). Further, the SS-PSE models without using imputed visibility would not converge in 6 of the 15 datasets (40%) and produced poor estimates in many other cases; for example, the Yerevan PWID datasets. Imputed visibility SS-PSE makes size estimation possible in cases where they could not be previously calculated, both for older studies where needed questions were not correctly asked on the survey and for cases where SS-PSE models without visibility fail to converge. In cases where these other PSE methods are possible, imputed visibility SS-PSE still performs favorably.

### Trend Analysis

**Figure 4:**
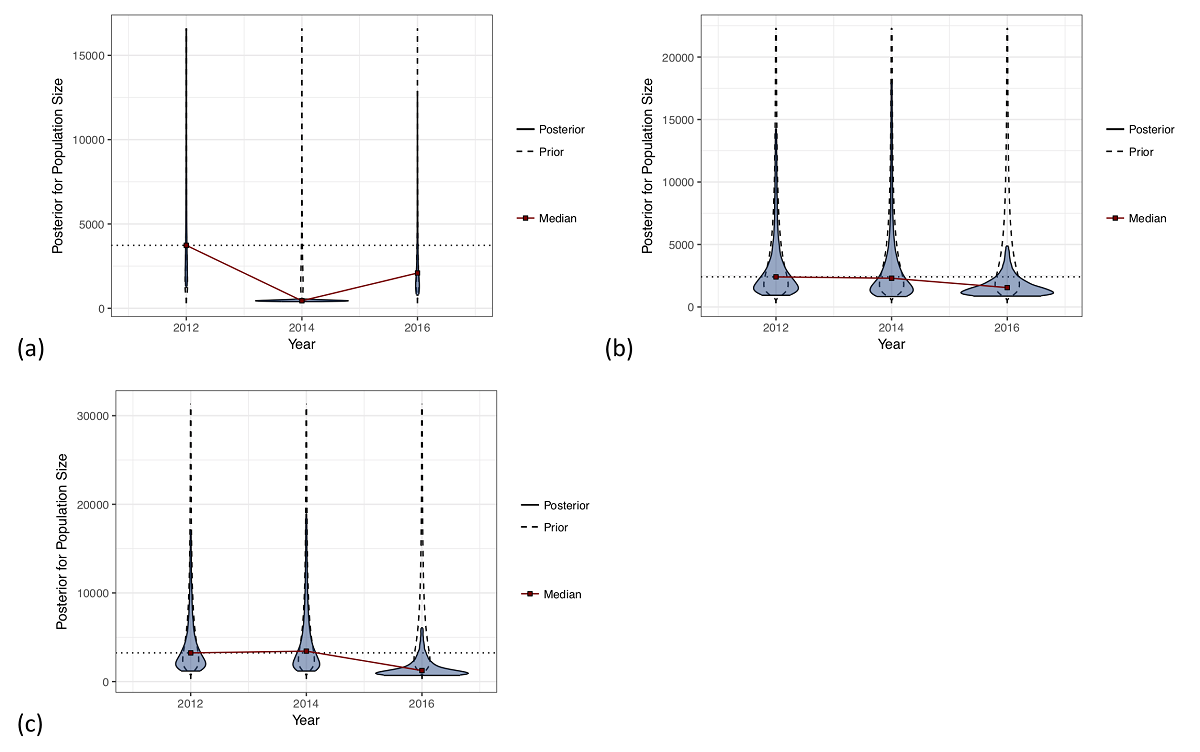
Trend analysis plots showing the prior and posterior distributions for 2012, 2014, and 2016 biobehavioral surveys in Yerevan of (a) female sex workers (FSW), (b) men who have sex with men (MSM), and (c) people who inject drugs (PWID). The median population size estimate is indicated by a red point for each year and the prior median for all three years is shown as a horizontal dotted line.

The data considered included three rounds—2012, 2014, and 2016—of BBS for FSW, MSM, and PWID in Yerevan, Armenia. Imputed visibility SS-PSE models were fit for each year using the same prior median population size for each population, based on consultation with local experts. We compared the size estimates, descriptively, over these three years for each population. We present a visual inspection of trend in population size over time, as three years of data are not enough to do a time series analysis and a hypothesis test for equality depends on assumptions that may not be met by the RDS sampling process. Figure 4 shows the mirrored prior and posterior distributions for each year, with lines connecting the posterior median of each year. The prior distributions were the same for each year for a particular population; the placement of the posterior relative to the prior distribution indicates whether the estimate is being increased or decreased relative to the prior distribution based on the data. Because of sampling error, any time we draw a new random sample from the same population, we may get a slightly different estimate. This is a natural phenomenon in sampling and not a cause for concern.

**Table 2:** Comparison of imputed visibility SS-PSE (successive sampling-population size estimation) posterior medians with other population size estimation methods for the 15 Armenia datasets.

Population	Expert values (n)			Object multiplier	Service multiplier	WOC^a^ (best mean)	SS-PSE median (no visibility)	SS-PSE median (visibility)
	Low	Median	High					
FSW^b^, Yerevan 2012	1500	3143	9900	N/A^c^	N/A	N/A	2041	3734
FSW, Yerevan 2014	1500	3143	9900	N/A	N/A	N/A	469	445
FSW, Yerevan 2016	1500	3143	9900	571	1283	1615	No fit^d^	2090
FSW, Gyumri 2016	165	351	1089	150	92	196	277	171
FSW, Vanadzor 2016	115	239	759	204	156	67	275	205
MSM^e^, Yerevan 2012	2420	4202	6667	N/A	N/A	N/A	No fit	2407
MSM, Yerevan 2014	2420	4202	6667	N/A	N/A	N/A	No fit	2299
MSM, Yerevan 2016	2420	4202	6667	749	8300	11,900	1121	1550
MSM, Gyumri 2016	176	306	485	3659	N/A	138	168	249
MSM, Vanadzor 2016	123	214	339	150	N/A	40	No fit	666
PWID^f^, Yerevan 2012	1667	5842	14,473	N/A	N/A	N/A	1245	3236
PWID, Yerevan 2014	1667	5842	14,473	N/A	N/A	N/A	1743	3435
PWID, Yerevan 2016	1667	5842	14,473	9000	N/A	19,342	997	1245
PWID, Gyumri 2016	167	584	1446	3000	6800	26	No fit	596
PWID, Vanadzor 2016	117	409	1013	3000	7000	198	No fit	477

^a^WOC: wisdom of the crowd estimates.

^b^FSW: female sex workers.

^c^N/A: not applicable. Information was not collected at the time the study was implemented that would enable calculation of these values.

^d^SS-PSEs without visibility imputation where the value is *No fit* indicate that the model would not converge.

^e^MSM: men who have sex with men.

^f^PWID: people who inject drugs.

Therefore, even if the size of, for example, the Yerevan MSM population remained exactly constant over the period from 2012 to 2016, we would expect to get different estimates each year due to sampling. We used the overall variability of the estimates, indicated by the 90% credible intervals, to assess how unusual any particular year’s estimate was, and if it was actually indicative of a trend.

Panels (b) and (c) in Figure 4 indicate that even though the yearly posterior medians are different, we should not interpret a strong trend from these data. It may be tempting to view the size of the MSM and PWID populations as decreasing. However, the change in median population size over time is small relative to the overall variability in the posterior distributions, so we would caution against a strong interpretation of trend. Additionally, the appearance of trend in the Yerevan PWID populations relies heavily on the drop-off seen in 2016; the potential issues regarding the PWID posterior distribution from 2016 were previously mentioned in the discussion of Figure 3. Since the estimates from 2012 and 2014 are relatively consistent, we would advise against drawing any conclusions that rely too heavily on the posterior distribution from 2016. Nevertheless, it is worth considering a potential trend when additional years of data are collected, such as the 2018 BBS in Yerevan. Panel (a) in Figure 4 contains the 2014 FSW estimate from Yerevan, which, as noted previously, resulted in a *Bad* estimate, possibly due to a bottleneck in recruitment that resulted in only a subpopulation estimate. We therefore caution against reading too much into this apparent trend, as the 2012 and 2016 estimates are similar.

## Discussion

### Principal Findings

Imputed visibility SS-PSE provides an estimate for the size of a hidden population using data already routinely collected in an RDS survey. Unlike many other PSE methods, imputed visibility SS-PSE relies on only one data source and can therefore be performed retroactively, after an RDS study has already been conducted. Further, the visibility imputation modification allows for potentially erroneously self-reported network sizes to be modeled, making the method more robust to misreporting, missing values, and extreme values. However, given the difficulty measuring a hidden population and the potential for biases at many levels, including undetected bottlenecks in the network structure of the population, problems with the RDS sample, and errors in the prior size estimations, some estimates may not make sense. It is therefore always important to assess the quality of the PSE, rather than treating it as innately correct. Diagnostic plots, such as the plots of social network size by enrollment date, are useful tools to assess RDS and SS-PSE assumptions. The posterior distribution should also be examined to assess possible issues with model fit, which could be indicated by a flat distribution or one with a spike at large values of N.

When fitting the imputed visibility SS-PSE model, prior belief about the population size is specified. For these 15 datasets we used the prior median, as this was the information available. It is also possible to use the first and third quartiles or other distribution summary measures, based on available knowledge. Prior values should be ascertained before fitting the model and not altered when an estimate does not make sense, in order to avoid introducing bias from the researcher. Instead, when the posterior distribution has the appropriate shape, but the estimate does not align with expert knowledge, it is advisable to engage additional stakeholders and examine the study in more detail. In this exercise, we found that the MSM populations in all three cities considered had inconsistencies with the expert prior values provided. The maximum low prior value was larger than the minimum high prior value, making a *Great* fit impossible. One possible explanation is the inclusion of transgender women in the MSM populations during these studies. Therefore, there could be differences between what experts perceive as the size of the population, based on who is an eligible member of that population, and what members of the population perceive. There could also be inconsistencies among different study participants, as some may include transgender peers in their accounting of personal network size, while others may not. Because of these difficulties, the transgender population was split apart from the MSM population for the 2018 BBS study.

To examine the sensitivity of the imputed visibility SS-PSE model fits to the choice of prior median, we fit each model with three different prior medians: the average of two expert values and each expert value individually. The average of the two expert values was the final prior median used for the models presented in the Results section. Using the other prior medians does not drastically change the PSE. Although the point estimates are slightly larger for the larger prior median and slightly smaller for the smaller prior median, the values are very similar given the overall variability of the distribution. Superimposed posterior distributions for SS-PSE fits using these three prior medians for each of the 15 datasets are provided in Multimedia Appendix 1.

Evaluating the results from the imputed visibility SS-PSE, as well as other PSEs used in conjunction with RDS (ie, unique object and service multipliers, wisdom of the crowds), is essential given that they are prone to biases, which may lead to unrealistic over- and underestimations. Many size estimation techniques can be used as part of each survey to triangulate and validate the most optimal size estimation [2,3,5,9,31]. Further validation of size estimations relies on expert input from many stakeholders, including governmental and nongovernmental organizations working with the population, persons directly involved with the sampling, and people with knowledge about statistics and epidemiology. These collaborative efforts are needed to explain biases and failures to meet assumptions in both the sampling and the population size methods.

### Conclusions

The imputed visibility SS-PSE method of PSE can be used with existing RDS data sources to obtain reasonable estimates when benchmarked against prior expert knowledge. We demonstrate the performance of this method on 15 datasets of FSW, MSM, and PWID populations from three waves of BBS studies conducted using RDS from three cities in Armenia. This is the first assessment of the modification to the imputed visibility SS-PSE methodology on such a large variety of data and the first to consider trend analysis for the same population over three time points. This is also the first presentation of how to interpret different outputs from SS-PSE in real data. These studies cover a variety of recruitment structures and sizes coming from nine different underlying social networks. The results provide examples of good model fits, where the RDS assumptions appear to be satisfied and the resulting posterior distributions are of the appropriate shape, and bad model fits, where the RDS assumptions appear to be violated in diagnostic plots or the posterior distributions depart greatly from expert opinions. We find that the imputed visibility SS-PSE model performs favorably compared to other PSE methods for these populations; these other methods have no basis on which to assess bias and often give impossibly large or small estimates or produce no estimate at all. Because SS-PSE does not rely on data from multiple surveys or census information, it is a valuable method of PSE. However, there are limitations to its use. If RDS assumptions are violated or there are issues with convergence in the model, results from SS-PSE should be interpreted with caution. To this end, we also provide guidance and suggested methods for goodness of fit to assess the SS-PSE methodology and the overall quality of the estimates. We recommend that SS-PSE be used in conjunction with other PSE techniques commonly used in RDS to ensure generation of the most accurate and acceptable size estimation.

## Appendix

Multimedia Appendix 1 Diagnostic and sensitivity plots.

## Abbreviations

AMD: Armenian Dram
BBS: biobehavioral surveys
FSW: female sex workers
MSM: men who have sex with men
PSE: population size estimation
PWID: people who inject drugs
RDS: respondent-driven sampling
SS-PSE: successive sampling-population size estimation
UNAIDS: The Joint United Nations Programme on HIV/AIDS
WOC: wisdom of the crowd estimate
