# Supplementary material for: Population Size Estimations Among Hidden Populations Using Respondent-Driven Sampling Surveys: Case Studies From Armenia
Source: JMIR Public Health Surveill. 2019 Mar 14;5(1):e12034. doi: 10.2196/12034 (PMC6437611; doi:10.2196/12034)
Supplement: Multimedia Appendix 1 [file publichealth_v5i1e12034_app1.pdf]

Recruitment Plots

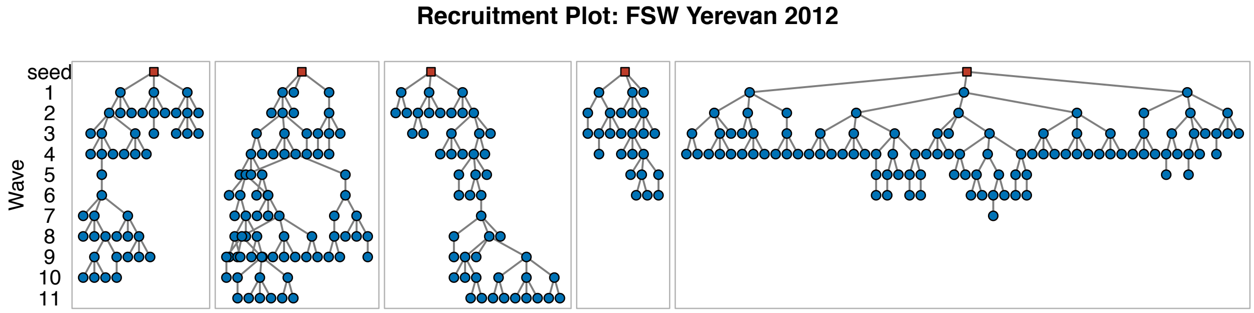

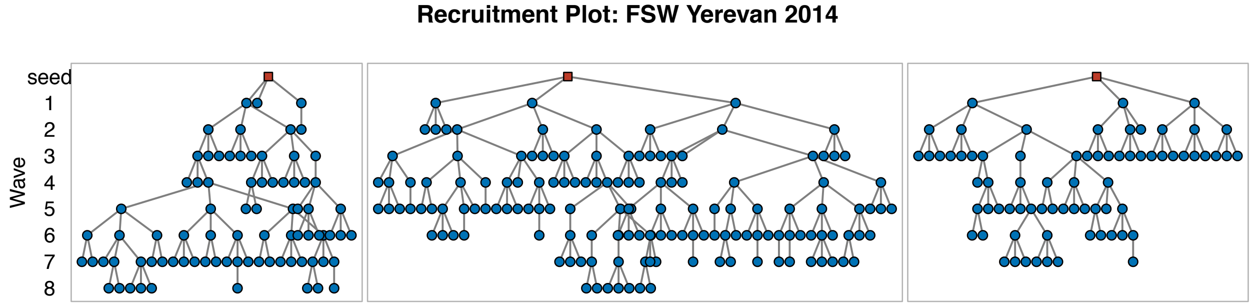

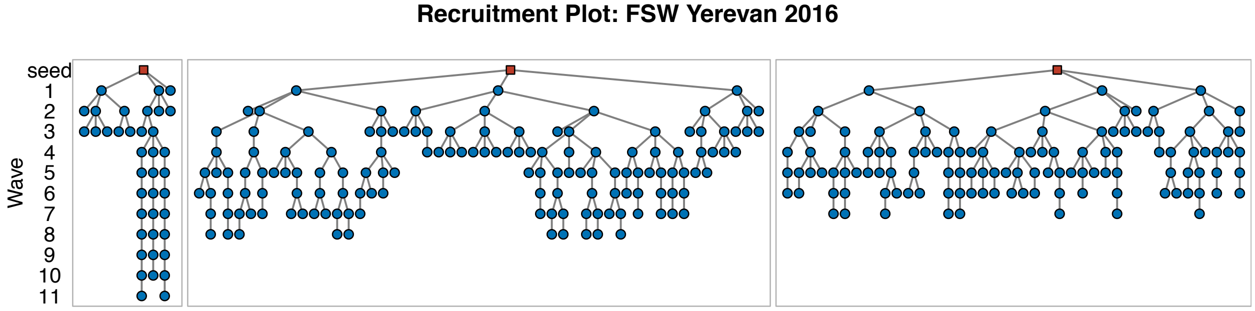

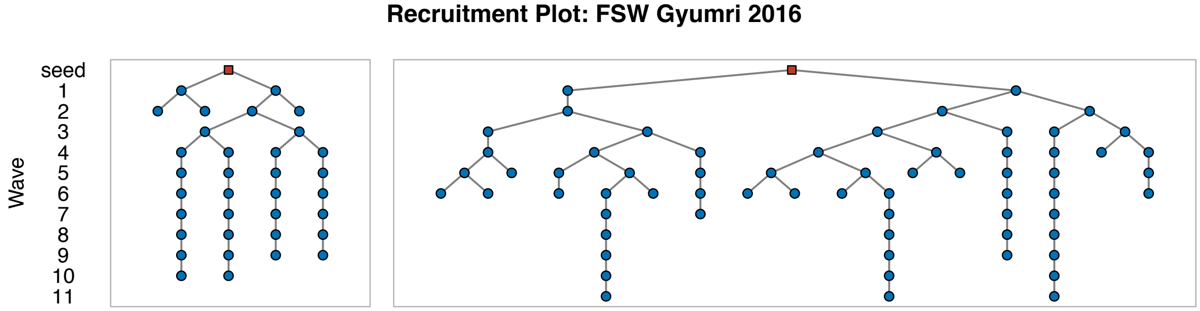

Recruitment Plot: FSW Vanadzor 2016

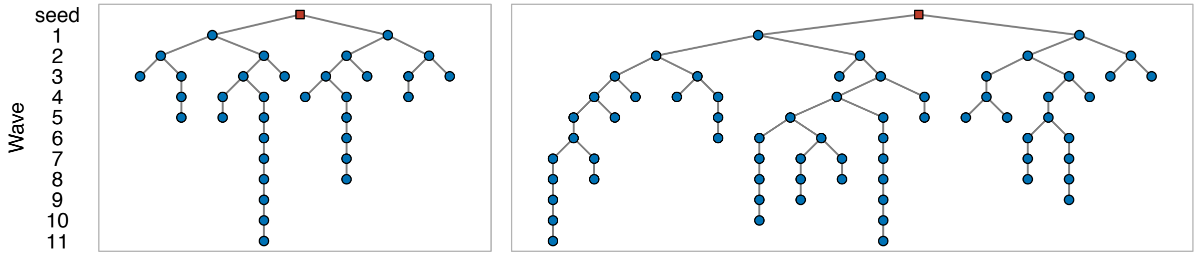

Recruitment Plot: MSM Yerevan 2012

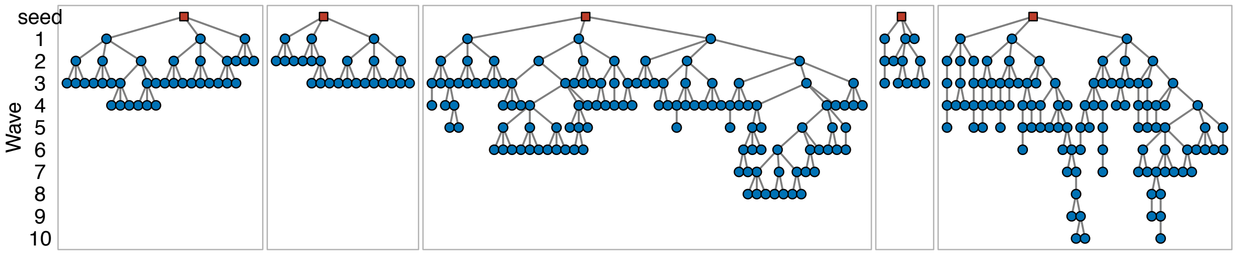

Recruitment Plot: MSM Yerevan 2014

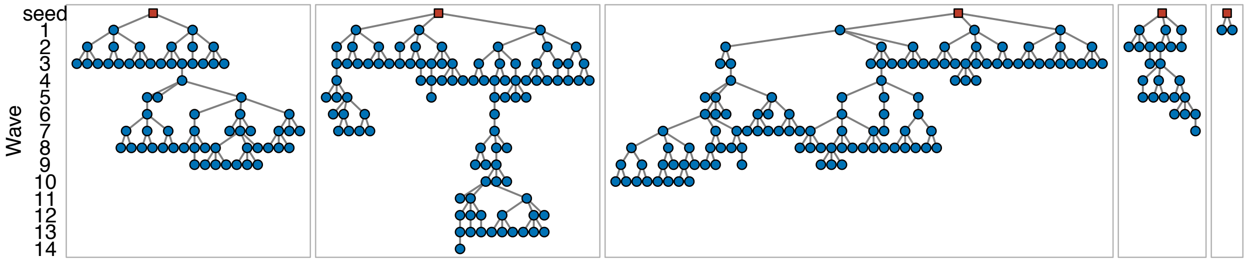

Recruitment Plot: MSM Yerevan 2016

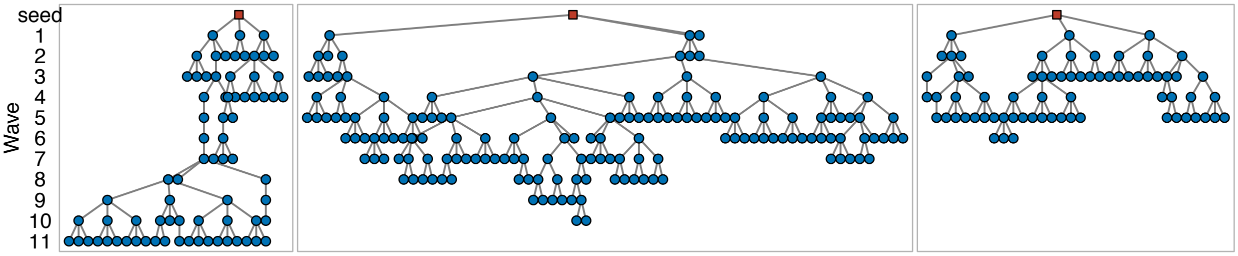

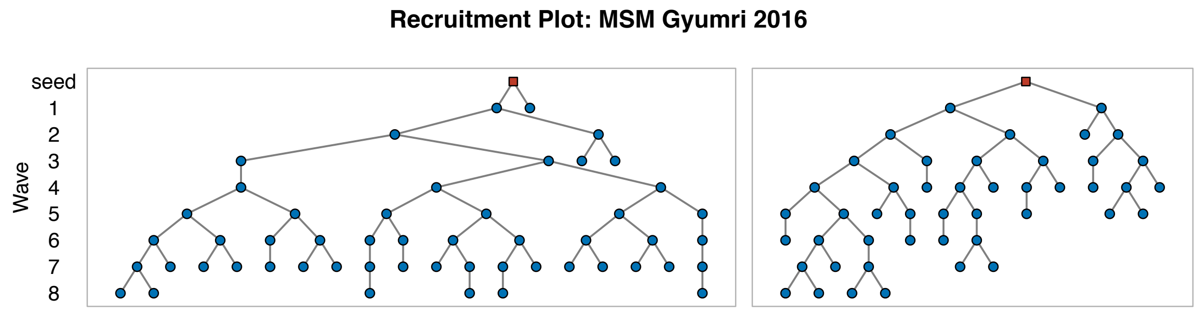
Recruitment Plot: MSM Gyumri 2016
Wave
seed
1
2
3
4
5
6
7
8

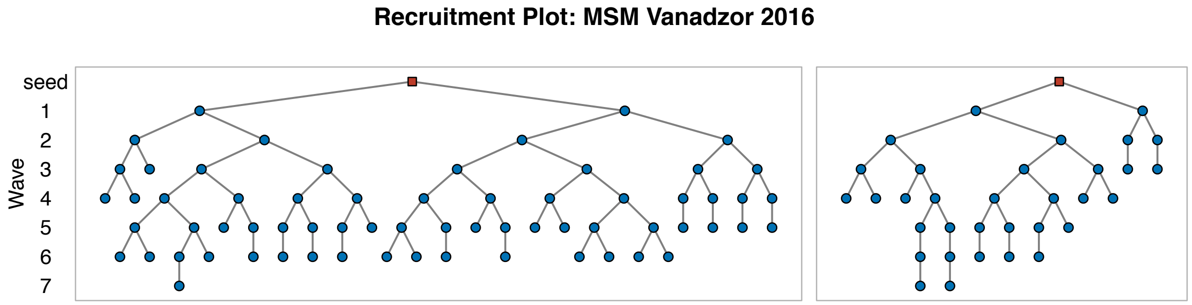
Recruitment Plot: MSM Vanadzor 2016
Wave
seed
1
2
3
4
5
6
7

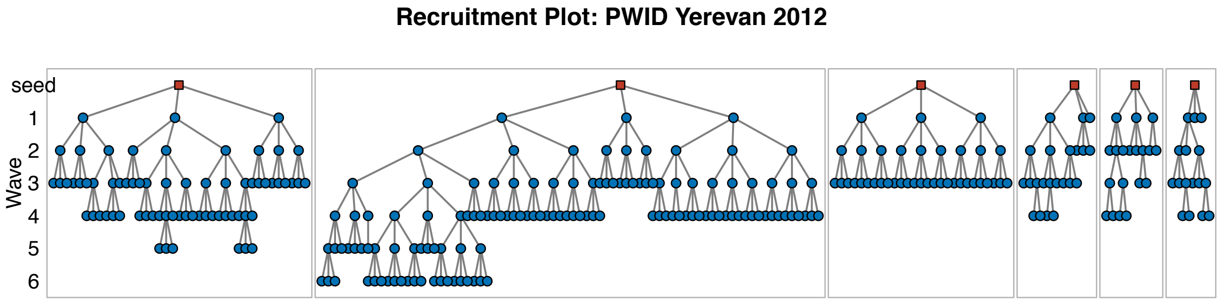
Recruitment Plot: PWID Yerevan 2012
Wave
seed
1
2
3
4
5
6

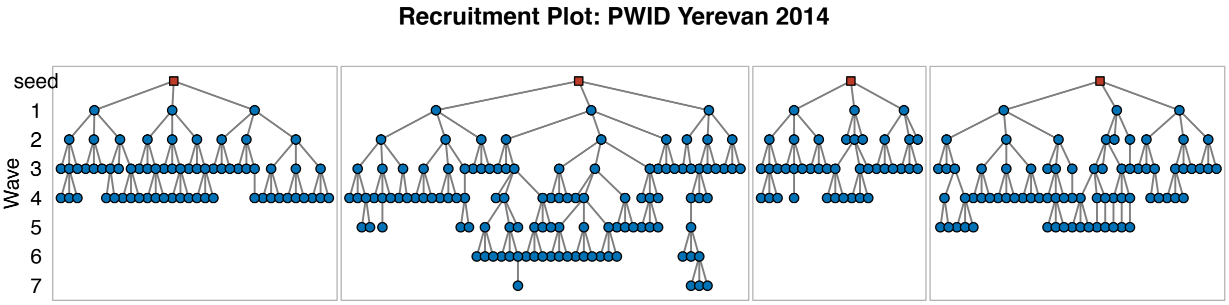
Recruitment Plot: PWID Yerevan 2014
Wave
seed
1
2
3
4
5
6
7

Recruitment Plot: PWID Yerevan 2016

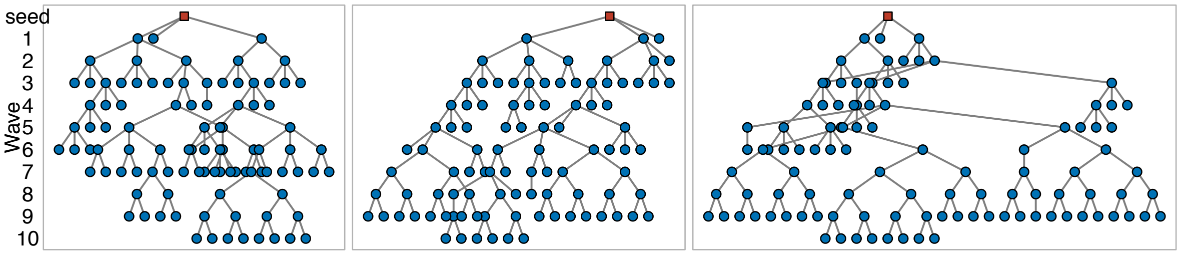

Recruitment Plot: PWID Gyumri 2016

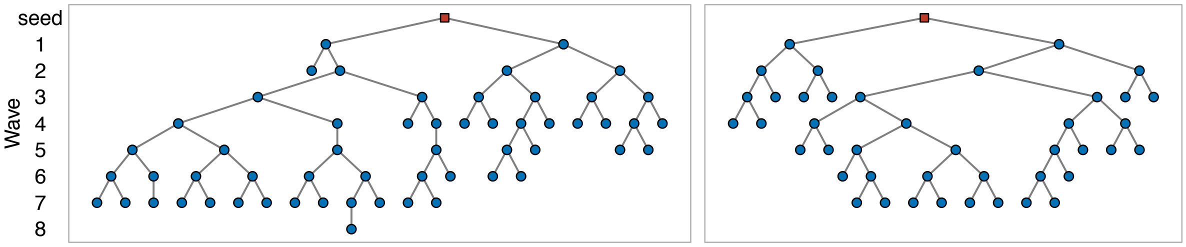

Recruitment Plot: PWID Vanadzor 2016

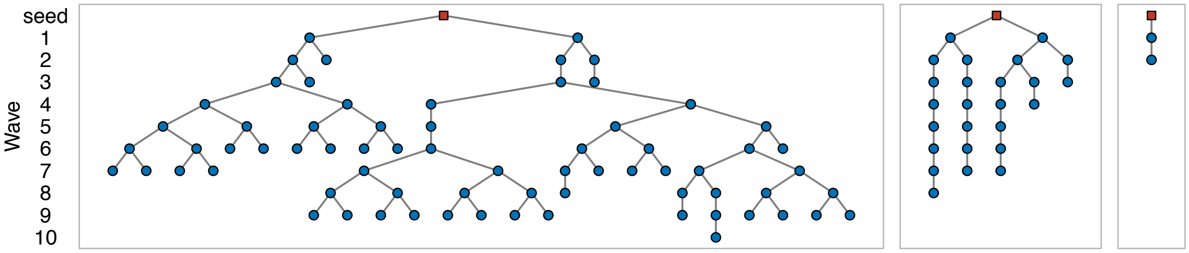

Reported Degree by Date of Enrollment

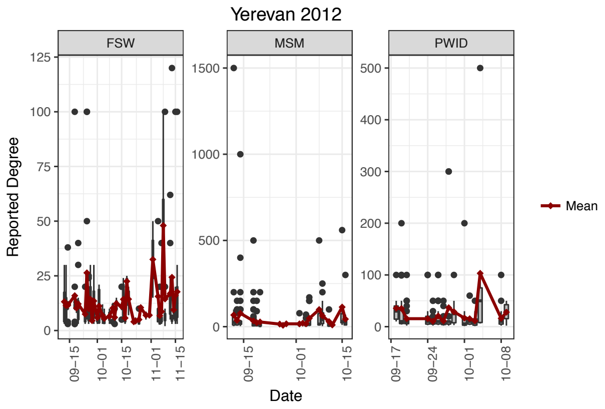

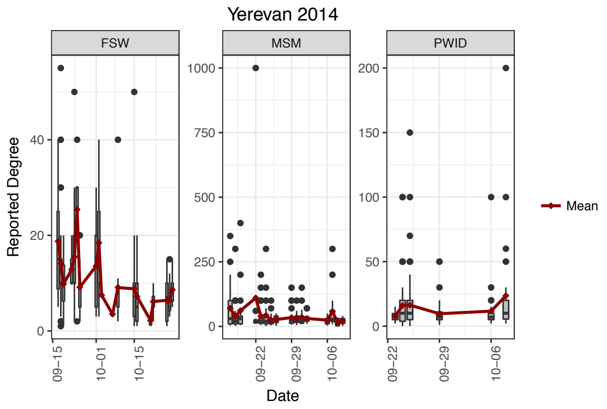

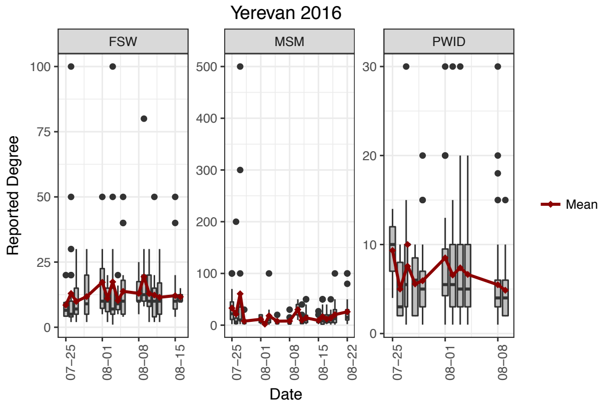

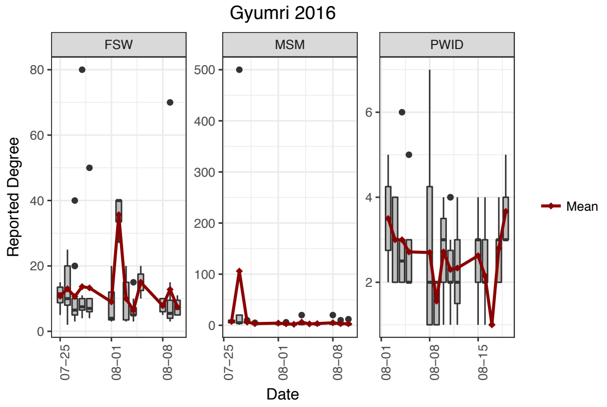

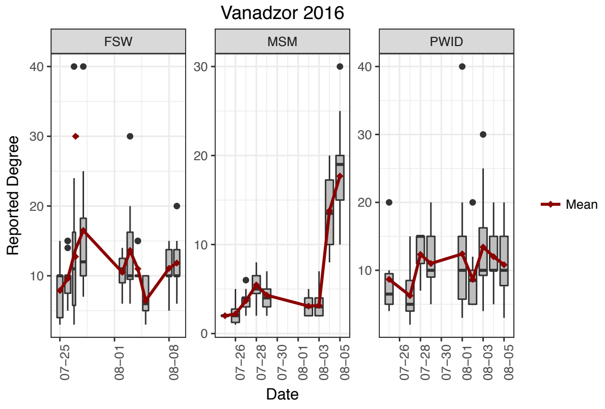

Posterior Distributions

FSW Yerevan 2012

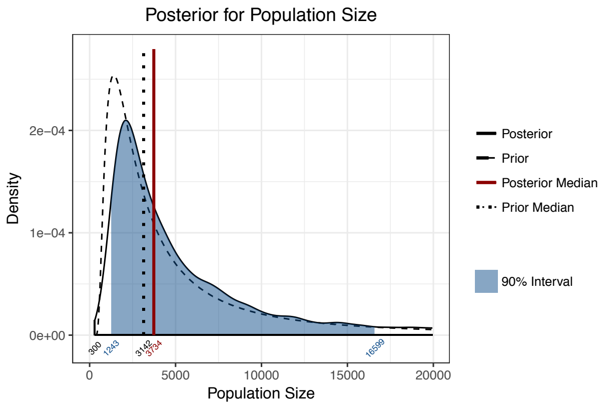

FSW Yerevan 2014

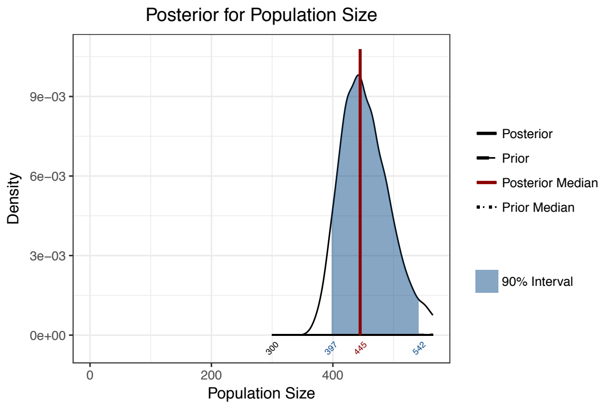

FSW Yerevan 2016

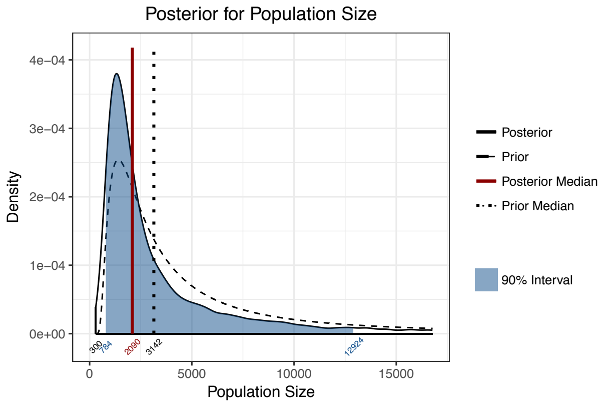

FSW Gyumri 2016

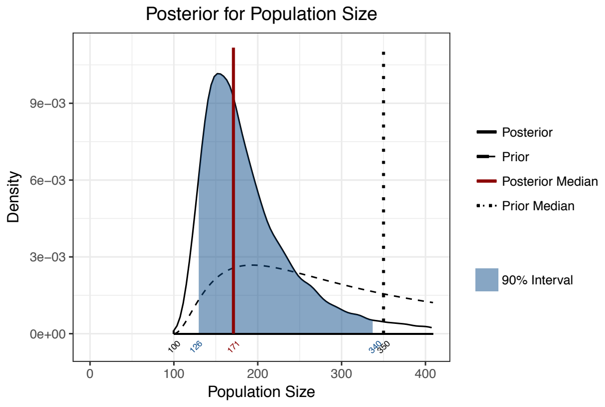

FSW Vanadzor 2016

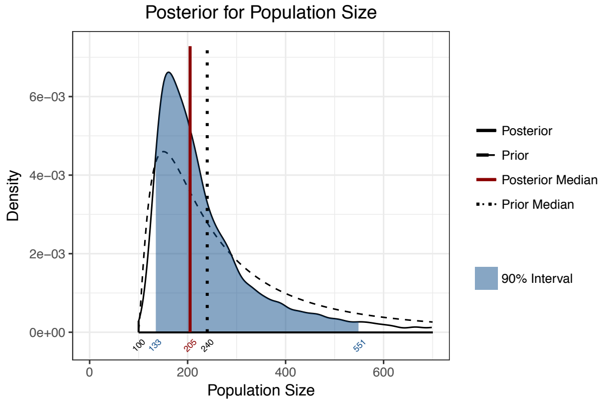

MSM Yerevan 2012

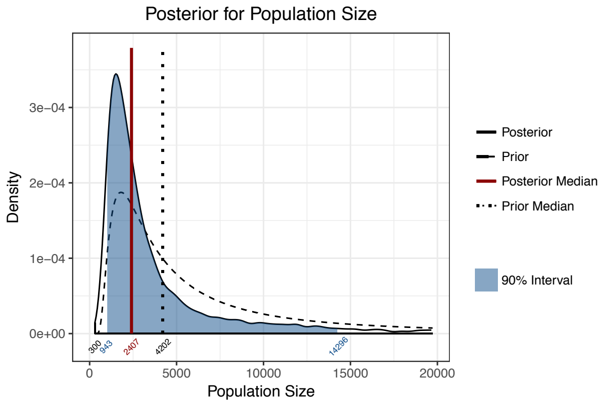

MSM Yerevan 2014

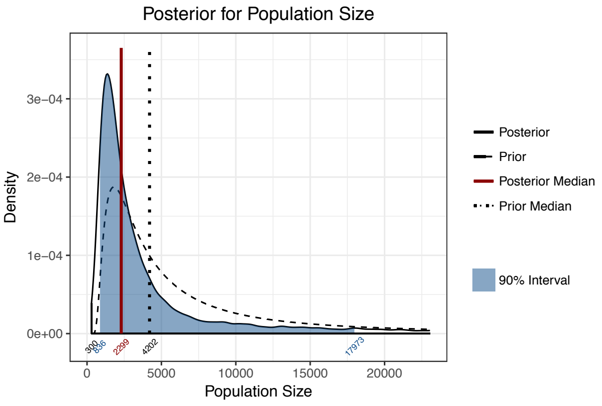

MSM Yerevan 2016

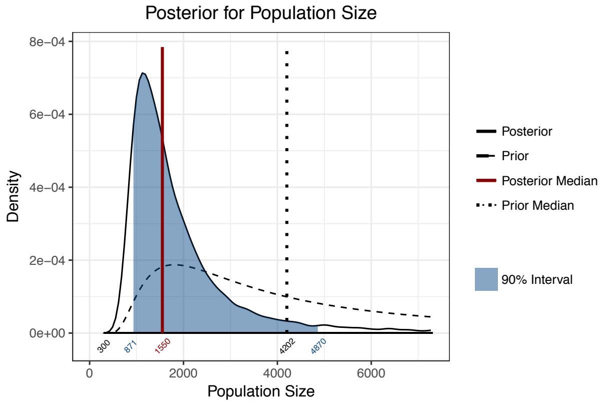

MSM Gyumri 2016

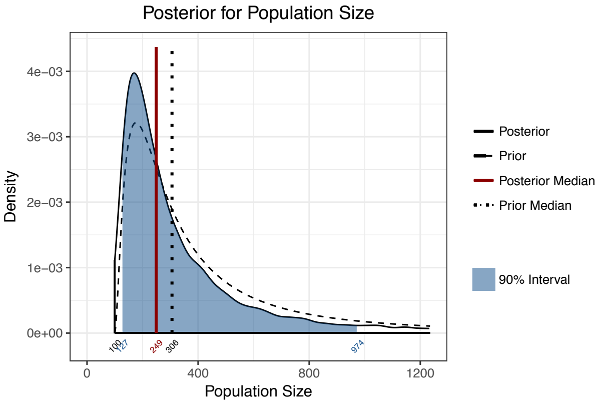

MSM Vanadzor 2016

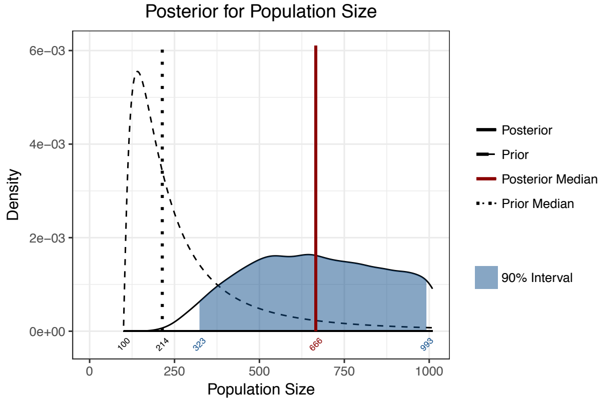

PWID Yerevan 2012

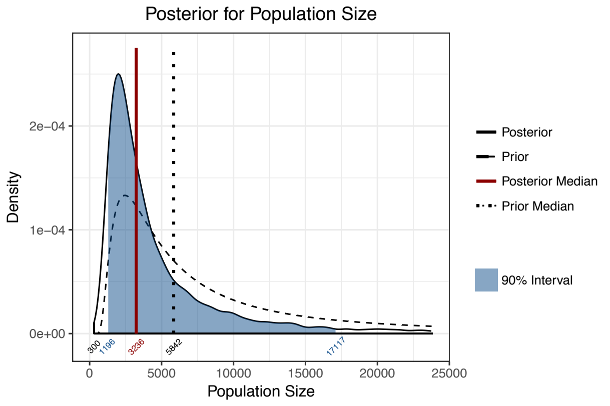

PWID Yerevan 2014

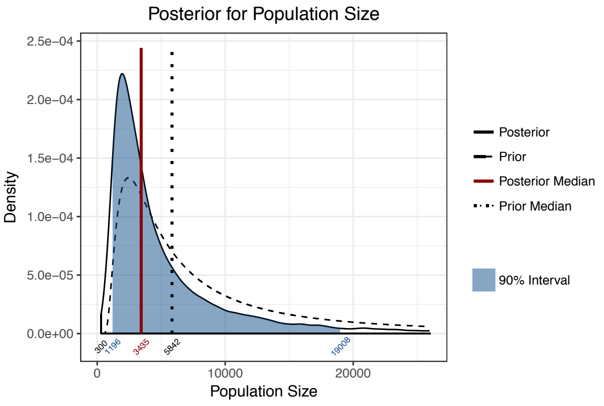

PWID Yerevan 2016

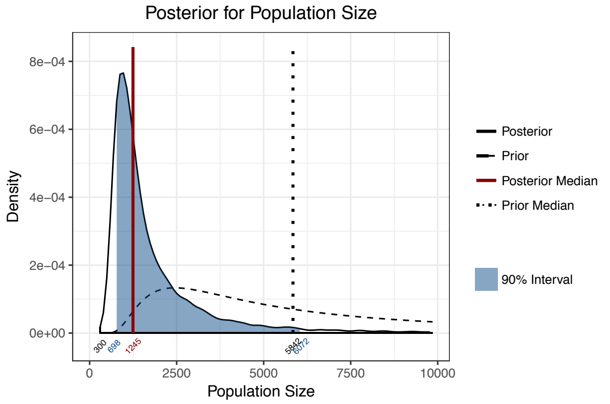

PWID Gyumri 2016

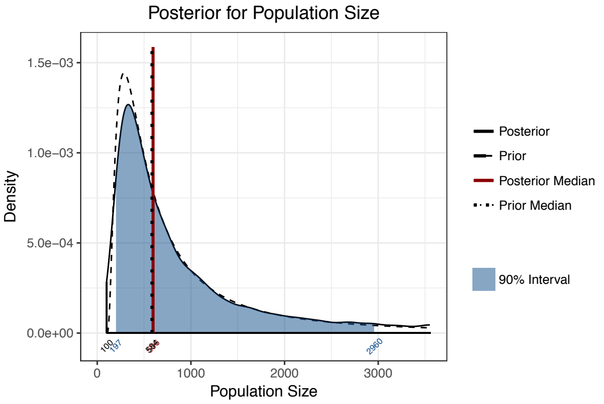

PWID Vanadzor 2016

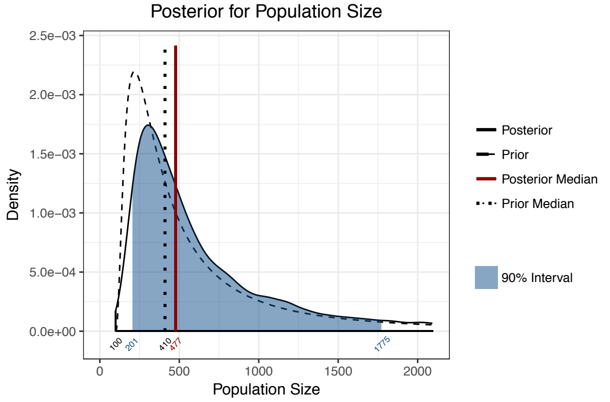

Sensitivity Analysis: posterior distributions using average of two expert medians (reg), lower of two expert medians (low), and higher of two expert medians (high). Vertical lines are posterior medians.

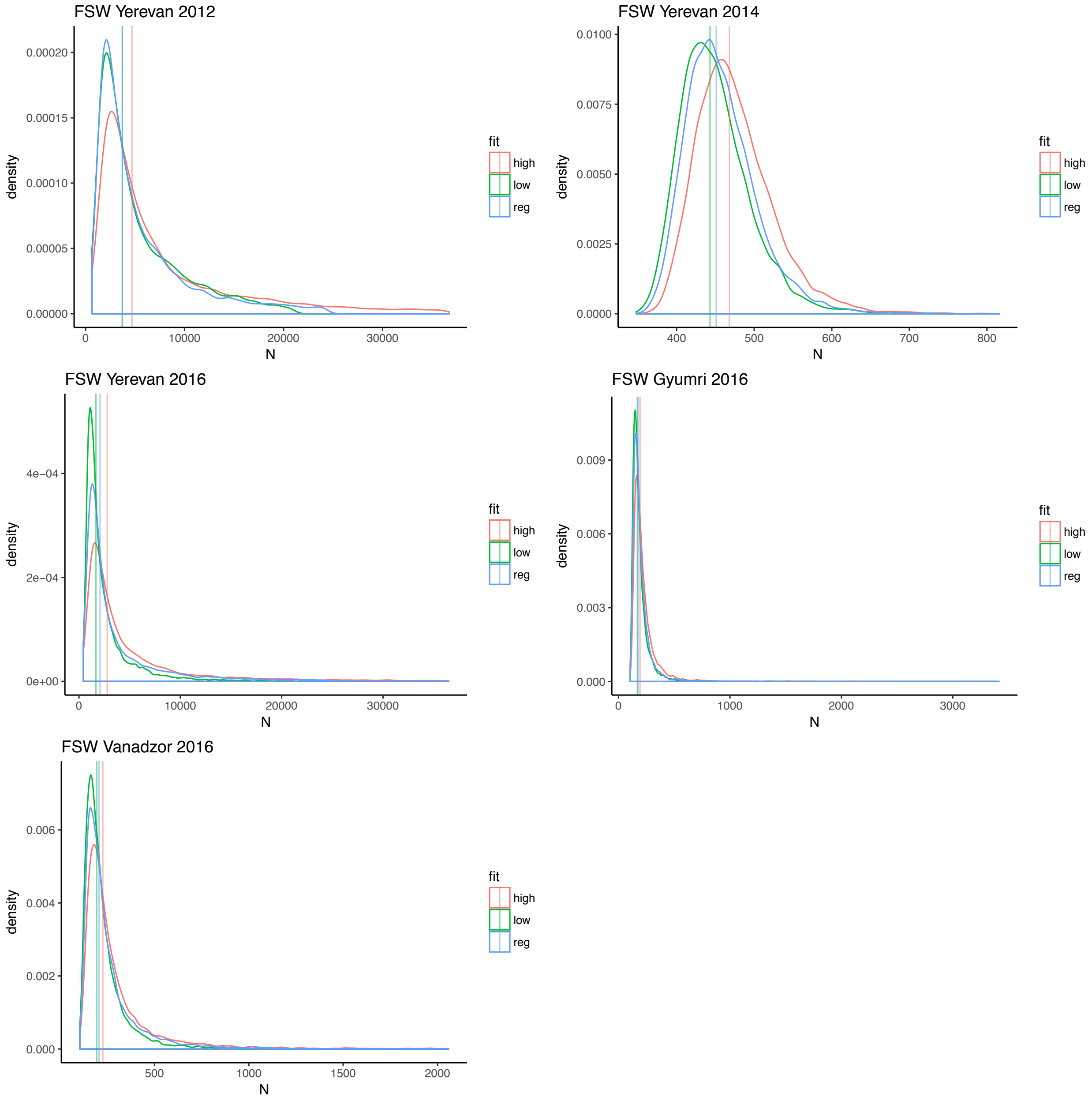

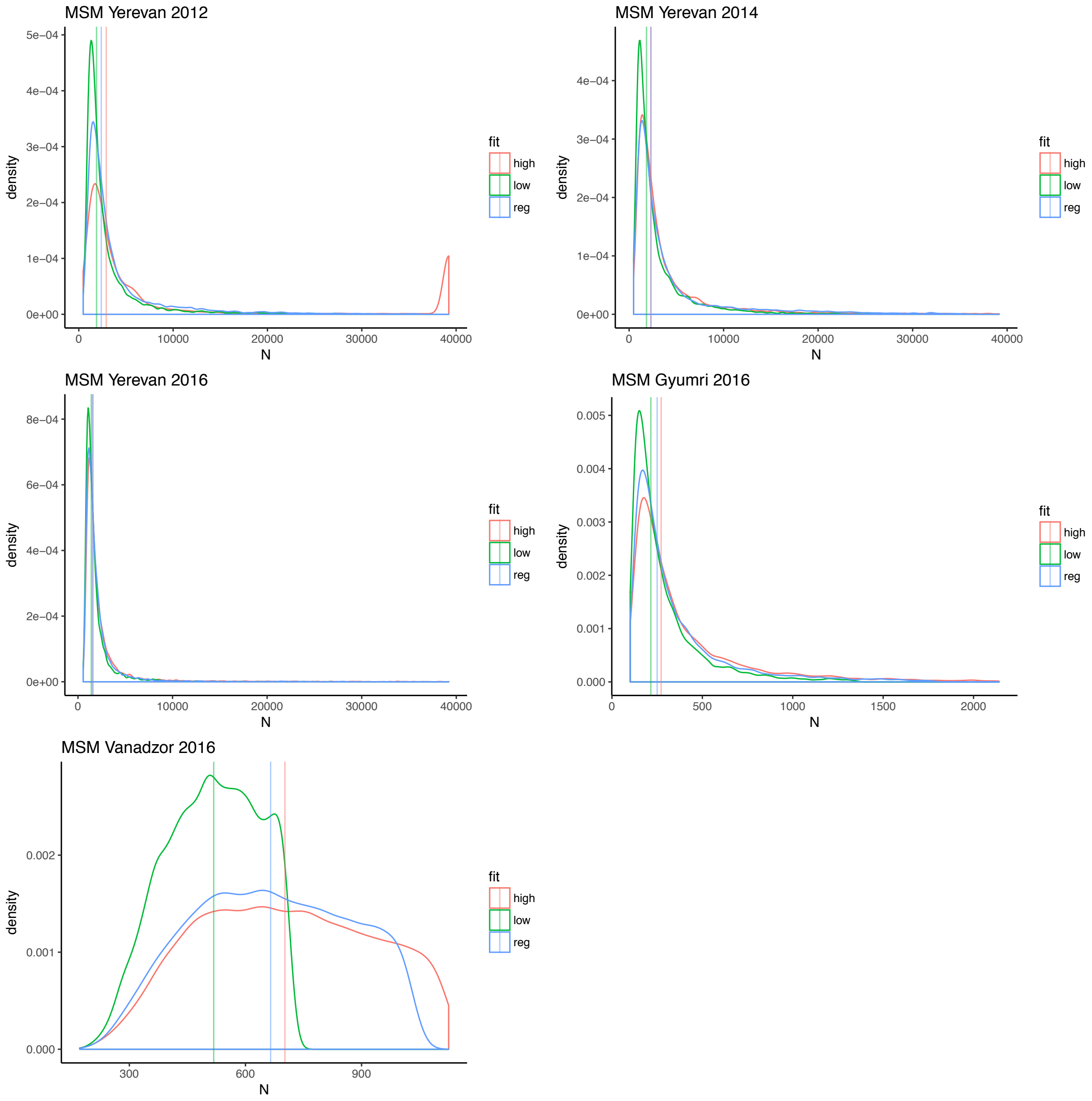
MSM Yerevan 2012
5e−04
4e−04
3e−04
2e−04
1e−04
0e+00
density
0
10000
20000
30000
40000
N
fit
high
low
reg
MSM Yerevan 2014
4e−04
3e−04
2e−04
1e−04
0e+00
density
0
10000
20000
30000
40000
N
fit
high
low
reg
MSM Yerevan 2016
8e−04
6e−04
4e−04
2e−04
0e+00
density
0
10000
20000
30000
40000
N
fit
high
low
reg
MSM Gyumri 2016
0.005
0.004
0.003
0.002
0.001
0.000
density
0
500
1000
1500
2000
N
fit
high
low
reg
MSM Vanadzor 2016
0.002
0.001
0.000
density
300
600
900
N
fit
high
low
reg

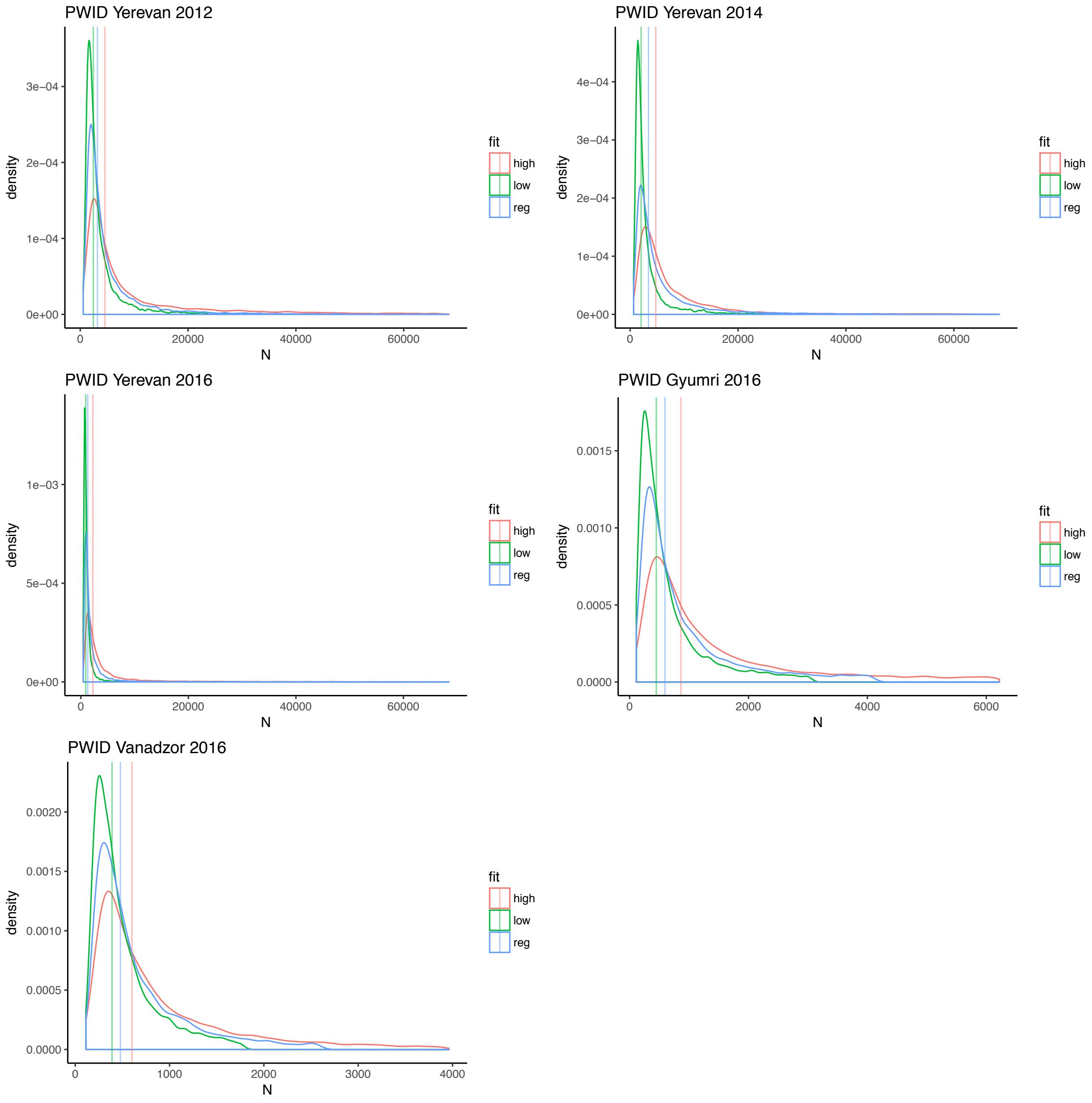
PWID Yerevan 2012
PWID Yerevan 2014
PWID Yerevan 2016
PWID Gyumri 2016
PWID Vanadzor 2016
density
N
fit
high
low
reg
0e+00
1e−04
2e−04
3e−04
4e−04
5e−04
1e−03
0.0000
0.0005
0.0010
0.0015
0.0020
0
1000
2000
3000
4000
6000
20000
40000
60000
